# A Membrane‐Based Strategy for the High‐Throughput Determination of Steroidal Hormones in Human Urine Using Natural Deep Eutectic Solvents Combined With Liquid Chromatography Coupled With Diode Array Detector

**DOI:** 10.1002/jssc.70229

**Published:** 2025-07-17

**Authors:** Lucas Morés, Camila Will, Eduardo Carasek, Josias Merib

**Affiliations:** ^1^ Departamento de Farmacociências Universidade Federal de Ciências da Saúde de Porto Alegre Porto Alegre Rio Grande do Sul Brazil; ^2^ Programa de Pós‐Graduação em Biociências Universidade Federal de Ciências da Saúde de Porto Alegre Porto Alegre Rio Grande do Sul Brazil; ^3^ Departamento de Química Universidade Federal de Santa Catarina Florianópolis Santa Catarina Brazil

**Keywords:** bioanalytical methods, estrogens, microextraction, sustainability, urine monitoring

## Abstract

Estrogen hormones are present in the human body at varying concentrations, either naturally or through ingestion. At regulated levels, these compounds perform vital functions in the body. However, elevated concentrations are related to deregulation of several processes, such as abnormal cell proliferation, which can lead to the development of cancer (breast and ovarian) and other diseases (endometriosis). In this work, an analytical methodology is proposed for the determination of 17β‐estradiol (E1), estrone (E2), and 17α‐ethinylestradiol (EE2) in human urine. Hollow fiber‐microporous membrane liquid–liquid extraction (HF‐MMLLE) is used with natural deep eutectic solvents (NADESs). This technique was associated with a 96‐well plate system followed by high‐performance liquid chromatography coupled with a diode array detector (HPLC‐DAD). The optimized conditions consisted of using thymol and camphor (1:1 v/v) as extraction solvent; an extraction time of 100 min; acetonitrile as desorption solvent; a desorption time of 30 min; and a sample pH adjusted to 12. Limits of quantification (LOQs) of 25 ng mL^−1^ for E2 and 50 ng mL^−1^ for E1 and EE2, and limits of detection (LOD) of 7.5 ng mL^−1^ for E2 and 15 ng mL^−1^ for E1 and EE2 were obtained. Intraday and interday precisions were lower than 8.7% and 21.6%, respectively. Relative recoveries were examined in two samples and ranged from 68.2% to 131.8%. The method was applied in samples from healthy volunteers, and concentrations from 75.6 to 182.7 ng mL^−1^ for E2 were found. Finally, the method was evaluated by two sustainable metrics to examine the green aspects of the experimental approach.

## 1 Introduction

The presence and concentration of specific hormones in the human body are critical indicators of several health conditions, including hormone‐related cancers. Among these compounds, steroid hormones, such as estrogens, play a vital role in regulating reproductive functions, bone health, cardiovascular systems, and neurophysiological processes. However, many studies have linked these compounds to the development of diseases, including endometriosis and breast and ovarian cancers [1, 2].

The primary natural estrogens in the human body include estrone (E2), 17β‐estradiol, and estriol. These hormones are mainly synthesized in the ovaries, with additional production in adipose tissue, liver, and adrenal glands. Estrone and estradiol are metabolized in the body to produce estriol. Among the metabolites, 17α‐estradiol and 17β‐estradiol (E1) are both produced from estradiol. Other products are also generated during metabolization pathways; however, the concentrations of bioactive metabolites are lower than the main estrogens. Additionally, synthetic estrogens such as 17α‐ethinylestradiol (EE2) are widely used in oral contraceptives and hormone replacement therapies due to their high stability and bioactivity. However, EE2 also poses significant concerns due to its persistence in the environment and potential endocrine‐disruption effect [1, 2, 3, 4]. In terms of biological activity, the most relevant hormones are E1, E2, and estriol, with E1 exhibiting the greatest biological activity, followed by E2, which exhibits approximately one‐third and 1% of its activity. Moreover, EE2 is metabolized by the body; however, its biological activity is greater than that of the metabolites produced in the biological process [1, 2, 3, 4].

Because of their effects, both natural and synthetic estrogens have been identified not only as critical biomarkers for health monitoring but also as emerging contaminants with significant environmental and public health implications. The accurate monitoring of these compounds in biological matrices is therefore essential for clinical diagnosis, therapeutic drug monitoring, epidemiological studies, and assessing exposure risks [2]. The persistent exposure to elevated levels of estrogens, particularly E1, E2, and EE2, has been linked to the course of hormone‐dependent diseases, such as breast and ovarian cancers. These hormones influence cellular proliferation, increasing the risk of DNA damage and tumor development in estrogen‐sensitive tissues. Therefore, the ability to monitor estrogen levels in biological fluids can provide valuable insights into early diagnosis and risk assessment [5, 6, 7, 8].

Urine, a noninvasive and readily available biological matrix, consists of suitable medium for detecting these hormones at low concentrations, enabling the application of bioanalytical methodologies for clinical and epidemiological studies [9]. Urine primarily consists of water and contains metabolites, proteins, salts, and other organic compounds. Despite being considered a challenging matrix, there are numerous sample preparation strategies that can permit the analysis of biomarkers using advanced analytical techniques [4, 10, 11, 12].

Bioanalytical methods are well‐established nowadays for a wide range of applications. However, a significant number of these methods are expensive, emphasizing the importance of developing alternative and efficient methodologies. In general, these methodologies are associated with sample preparation steps typically comprising extraction techniques. Sample preparation techniques aim to isolate the analytes and concentrate them to suitable levels for the detection and quantification, as well as making the sample compatible with the analytical instrumentation [13, 14]. Some classical techniques such as solid‐phase extraction (SPE) and liquid–liquid extraction (LLE) are highly employed; however, despite their applications and high efficiency, these techniques exhibit some issues including large consumption of toxic solvents, long extraction times, and extensive manual labor, among others. These limitations are not in accordance with the principles of green analytical chemistry (GAC) and the growing demand for sustainable laboratory practices [15, 16].

Microextraction techniques such as solid‐phase microextraction (SPME) and liquid phase microextraction (LPME) were introduced to overcome some of these limitations. Within LPME, different formats and configurations have been proposed, including hollow fiber‐LPME (HF‐LPME). A further development of HF‐LPME is the hollow fiber‐membrane microporous liquid–liquid extraction (HF‐MMLLE) [17]. In this regard, the extraction is performed using a solvent immobilized into the pores of a membrane. After the extraction, a liquid desorption step is performed followed by instrumental analysis. The experimental workflow is simple and straightforward to implement. Another advantage is that a support can be used for the membrane, so it can be coupled to a 96‐well plate system. Regarding the solvents that can be used into the pores of membrane‐based LPME techniques, alternatives such as the use of deep eutectic solvents (DES) are gaining significant attention [18, 19, 20, 21, 22].

Deep eutectic solvents (DES) are an emerging class of sustainable solvents formed by mixing two or more components, usually a hydrogen bond donor (HBD) and a hydrogen bond acceptor (HBA), which interact to produce a mixture with a melting point significantly lower than that of the individual constituents. These solvents exhibit low volatility, high thermal stability, and the ability to dissolve a wide range of organic and inorganic compounds. Additionally, their preparation is simple, and compounds with reduced toxicity and biodegradability can be obtained, consisting of promising alternatives for applications in green chemistry, such as extraction, catalysis, electrochemistry, and sample preparation [23, 24, 25].

Natural deep eutectic solvents (NADESs) were reported in 2011 by Verpoorte and collaborators [25]. The components that form the eutectic mixture are natural constituents, and the HBAs usually comprise ammonium chloride and choline chloride, or amino acids (alanine, proline, glycine, betaine). Related to the HBDs, organic acids (oxalic, lactic, malic, etc.) or carbohydrates (glucose, fructose, maltose, sucrose, sorbitol, xylitol, among others) are used. They are biocompatible and environmentally friendly, which agrees with GAC principles. The properties and sustainable aspects make these solvents interesting alternatives for sample preparation techniques, including membrane‐based approaches such as HF‐MMLLE [26, 27, 28, 29, 30, 31, 32].

In recent years, the assessment of the sustainability of analytical methodologies has become increasingly important, especially in sample preparation, which often represents the most waste‐generating step in analytical workflows. In this regard, several metrics have been developed to systematically evaluate the greenness of sample preparation techniques. Analytical Greenness Calculator for Sample Preparation (AGREEprep) is a semiquantitative metric based on a circular pictogram divided into segments, offering a visual and numerical score that facilitates the comparison among different methods. It considers factors such as solvent type and amount, energy consumption, automation, miniaturization, and waste generation [33]. Complex Green Analytical Procedure Index (ComplexGAPI) extends the capabilities of the original GAPI tool by providing a comprehensive pictogram that visualizes the greenness across the entire analytical process, with particular emphasis on the sample preparation step. It allows for a qualitative and comparative assessment of environmental impact based on color‐coded symbols that reflect the eco‐friendliness of each step [34].

In this study, for the first time, an analytical methodology combining the use of NADESs and HF‐MMLLE was proposed for the determination of the steroidal hormones E1, E2, and EE2 in human urine followed by high‐performance liquid chromatography coupled with a diode array detector (HPLC‐DAD). Additionally, this experimental workflow was associated with a semiautomated 96‐well plate system that enhanced the sample throughput. The analytical methodology was fully optimized and applied for analyzing real samples, and the sustainable aspects of the method were examined using ComplexGAPI and the AGREEprep.

## 2 Methodology

### 2.1 Reagents and Materials

Analytical standards of the hormones 17β‐estradiol (E1) (≥98%), estrone (E2) (≥98%), and 17α‐ethinylestradiol (EE2) (≥98%) were obtained from Sigma Aldrich (St. Louis, MO, USA). Acetonitrile (ACN), methanol (MeOH), ethyl acetate (EtAc), and acetone (CET) solvents were purchased from Merck (Kenilworth, NJ, USA) at HPLC purity grade. Ultrapure water (UPW) (18.2 MΩ cm^−1^) was obtained by Mega Purity purification system (Billerica, MA, USA). The following natural compounds were evaluated: butyric acid (≥99%), hexanoic acid (≥98%), octanoic acid (≥98%), nonanoic acid (≥98%), decanoic acid (≥98%), dodecanoic acid (≥99%), levulinic acid (≥98%), thymol (≥98.5%), and camphor (≥96%), all obtained from Sigma Aldrich. Membranes of polypropylene (Accurel PP300/1200) (internal diameter 1.2 mm, thickness 300 mm, and pore size 0.2 mm) were obtained from Membrane (Wuppertal, Germany). Sodium hydroxide for pH adjustment was obtained from Vetec (Rio de Janeiro, RJ, Brazil).

### 2.2 Instrumentation and Chromatographic Conditions

A 96‐well plate system was obtained from PAS Technologies Inc. (Bruder Mannesmann Werkzeuge, Remscheid, Germany). Wells with a volume of 500 µL were used for solvent impregnation and desorption step of the membranes, and 2 mL wells were used for the extraction step. A liquid chromatograph (model LC‐20AT) with diode array detector (model SPD‐20A Series) coupled with a Rheodyne 7725i manual injector (Rohnert Park, CA, USA) and an injection loop of 20 µL obtained from Shimadzu (Kyoto, Japan) was used. The mobile phase flow rate was 1 mL min^−1^ with ACN (A) and UPW (B) under the following conditions: 0–6 min with 35% A; 6–9 min increased to 80% A; 9–15 min the initial condition was reestablished. The wavelength used for the analysis was 200 nm. Chromatographic separation was performed in reversed phase with a Zorbax Eclipse XDB‐C18 column (Agilent, 250 mm × 4.6 mm and particle size 5 µm). A centrifuge model 80–2b 12 tubes/15 mL (Centrilab, Brazil) for centrifugation of the urine samples was used.

### 2.3 Preparation of NADES and Method Optimization

Initially, the NADES were produced according to a procedure previously reported in literature [35]. Regarding the HF‐MMLLE procedure, polypropylene membranes were prepared by cutting into 1 cm, washing them in ACN and then in acetone, followed by allowing the solvent to dry at room temperature. After cleaning, the membrane pieces (1 cm) were attached to the metallic pins of a 96‐well‐plate system. The NADES previously prepared were immobilized only into the membrane pores for 10 min under agitation. Regarding the optimization step of the HF‐MMLLE approach, a blank urine sample obtained from a male volunteer was used. For the extraction step, 1.5 mL of sample was added to the 2‐mL wells of a Teflon plate in the 96‐well plate system. Subsequently, a desorption step was performed using 300 µL of solvent in a separate set of 500‐µL wells. No contamination or carryover effect was observed, as the membranes were used only once.

Urine samples were kept under refrigeration at 4°C in PFTE flasks and subjected to centrifugation before the analysis. The parameters optimized were type of desorption solvent (through a simplex lattice design) utilizing ACN, MeOH, and EtAc; desorption time through a univariate strategy employing 10, 20, and 30 min; extraction time (17–110 min); and pH of the sample (5–13) through a central composite design. Data treatment was performed using Statistica 8.0 and Microsoft Excel 2010 software.

### 2.4 Analytical Parameters of Merit

Analytical parameters of merit were obtained through a five‐level calibration curve from extractions in a blank urine sample obtained from a volunteer. Limits of detection, limits of quantification (LOQs), coefficients of determination (*r* ^2^), and linear ranges were determined. Intraday precision (*n* = 3) and interday precision (*n* = 9) assays were also assessed. In addition, relative recoveries were evaluated to determine the accuracy of the method in different urine samples using three concentrations. Urine of healthy female volunteers, who make use of contraceptives, was used to evaluate the applicability of this method.

### 2.5 Greenness Evaluation

AGREEprep and ComplexGAPI were used to assess the greenness of the methodology developed in this study. Regarding AGREEprep, sample preparation aspects such as sampling, reagents, solvents, materials, instrumentation, waste, economics, and analyst safety are considered. In this regard, a score from 0 to 1 is generated. A greener method is obtained when the global score is closer to 1; on the other hand, a less sustainable method is achieved with global scores closer to 0 [33]. ComplexGAPI is more comprehensive and related to the complete analytical methodology, consisting of a larger hexagon (comprising five pentagons) and one smaller hexagon. This strategy considers the parameters of instrumentation, reagents, solvents, purification, research, and economy. Therefore, different colors are employed, including green for a low environmental impact, yellow for a medium environmental impact, and red for a high environmental impact [34].

## 3 Results and Discussion

### 3.1 Production and Selection of NADES

The NADESs evaluated in this study were produced according to Table S1. The choice of the best solvent was performed in UPW spiked with 500 ng mL^−1^ of the analytes. All NADESs described in Table S1 were used, and the extraction was performed for 90 min using 1.5 mL of UPW, pH adjusted to 11, followed by desorption performed for 20 min in ACN. The results are shown in Figure S1 in which the normalized chromatographic peak area of each analyte was used as analytical response.

**FIGURE 1:**
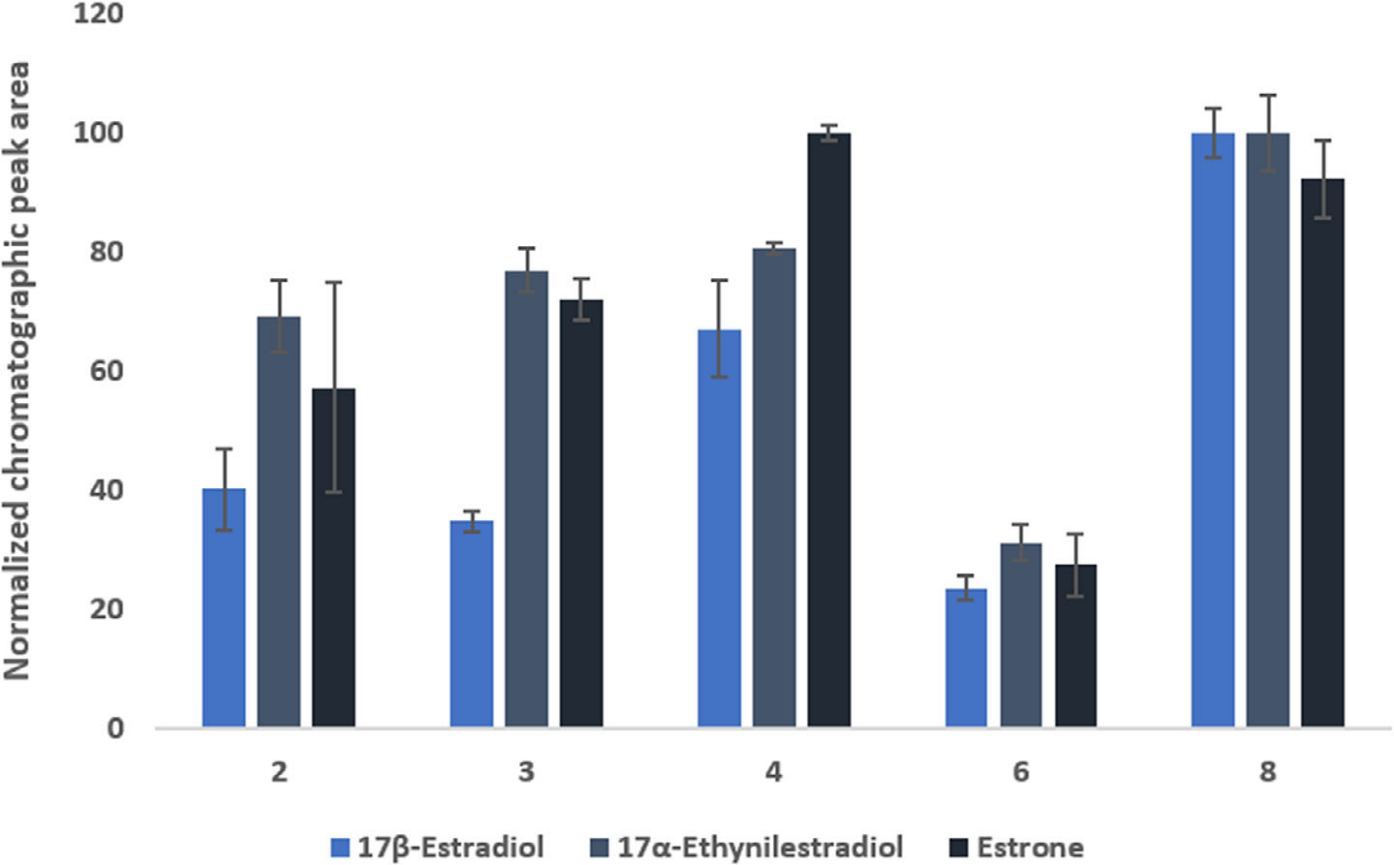
NADES evaluated for the extraction of steroidal hormones from urine samples, as follows: (2) Thy:C10; (3) Ca:C10; (4) L:Thy; (6) C12:C6; (8) Thy:Cam. Experimental conditions: urine adjusted to pH 10; extraction time of 60 min; desorption time of 20 min; acetonitrile as desorption solvent; analytes spiked at 500 ng mL^−1^.

It is possible to observe that poor results were obtained for the NADES 1, 7, and one analyte was not able to be extracted with NADES 9. The NADES 5 exhibited good responses; however, due to C12 and C9 within, the chemical structure this compound was prone to crystallize at room temperature, which significantly hindered the applicability of this solvent. Related to the NADES 10, satisfactory response was obtained for the extraction of EE2; however, another crystallization issue was observed involving C10. Therefore, the NADES 2, 3, 4, 6, and 8 were selected for a more detailed investigation of the extraction efficiency. Additionally, membrane without any solvent (11) was employed to examine some possible capacity of extracting the analytes. This result has shown responses lower than 10% compared to those obtained with the best NADES, indicating that the solvent plays a crucial role in the extraction of the compounds. The results obtained with the NADES are shown in the bar graphs of Figure 1 using the normalized areas of the chromatographic peaks.

According to Figure 1, the best responses were obtained using the NADES 8 comprising thymol and camphor that was liquid at room temperature. Therefore, in addition to its extraction efficiency for the hormones, the viscosity of this solvent permitted a simple procedure to fill the pores of polypropylene membranes. Additionally, the chemical structure of the constituents allowed for different interactions with the analytes, including hydrogen bonding and van der Walls which may have influenced the satisfactory performance of this NADES. On the other hand, the proportion between the two compounds to produce NADES was not evaluated, as the responses obtained using the equimolar ratio were satisfactory for the development of the proposed methodology.

### 3.2 Optimization of HF‐MMLLE Procedure

**FIGURE 2:**
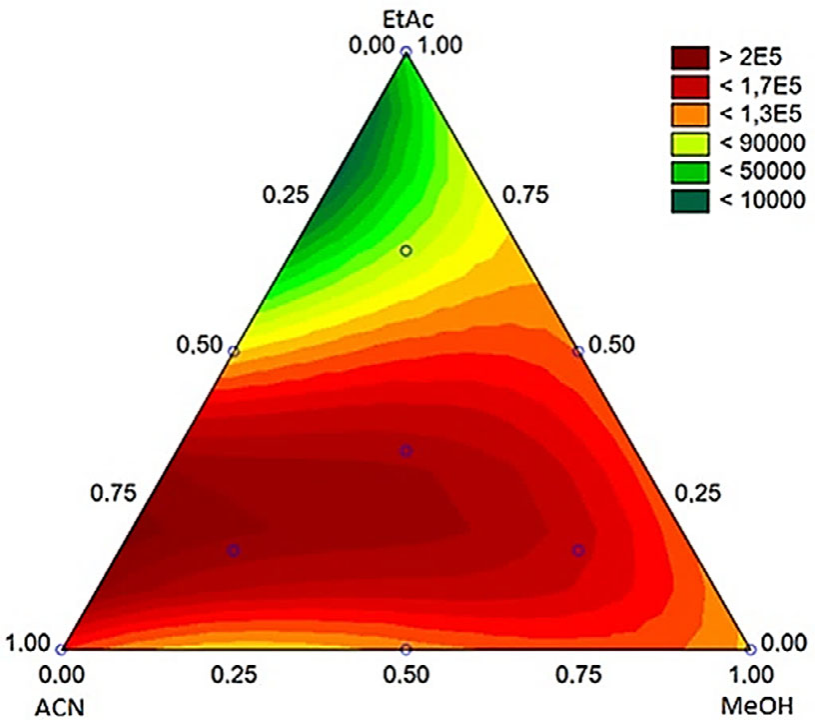
Response surface obtained for the optimization of desorption solvent. Experimental conditions: urine adjusted to pH 11; extraction time of 60 min; desorption time of 20 min; analytes spiked at 500 ng mL^−1^; NADES—Thy:Cam (1:1 v/v).

First, desorption solvent was optimized to permit a satisfactory solubilization of the NADES impregnated into the membrane pores. Therefore, ACN, MeOH, and AcEt were examined through a ternary surface obtained through a Simplex Lattice design. The result of this optimization is shown in the response surface of Figure 2 considering the geometric means of the chromatographic peak area for the analytes.

The response surface of Figure 2 exhibited *R* ^2^ = 0.974 for a cubic model, and the best analytical response was obtained with 75% acn and 25% EtAc. However, to achieve better peak shapes for the analytes, 100% ACN was considered optimized condition. This condition permitted a significant improvement in peak shapes being beneficial for separation.

Afterward, desorption time was studied through a univariate planning using 10, 20, and 30 min. The normalized means of the chromatographic peaks of the analytes were considered analytical responses, and the results are shown in the bar graph of Figure S2. It is possible to observe that the maximum is obtained with 30 min of desorption for the three analytes. Therefore, 30 min was maintained for further evaluations.

**FIGURE 3:**
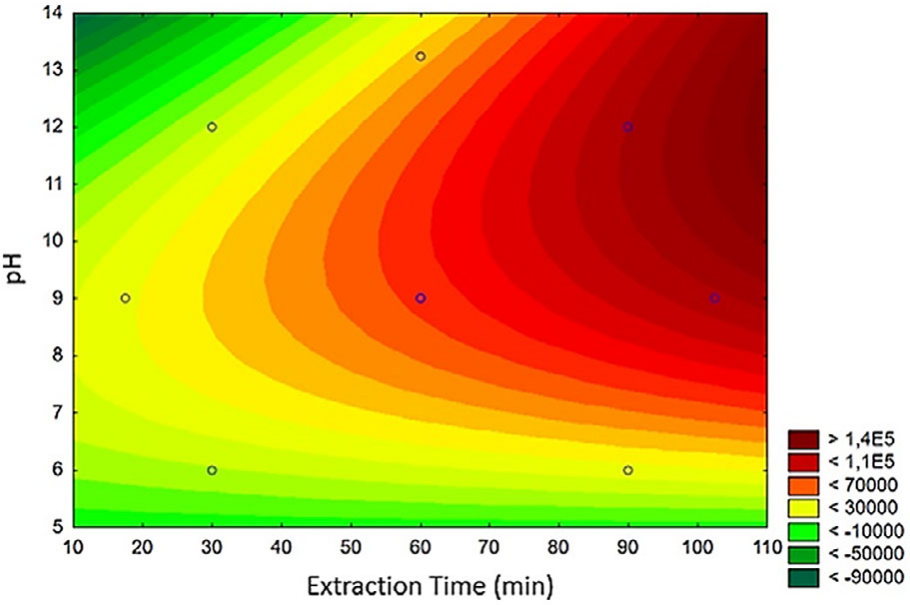
Response surface obtained for the optimization of extraction time and sample pH. Experimental conditions: desorption time of 30 min; acetonitrile as desorption solvent; analytes spiked at 500 ng mL^−1^; NADES—Thyl:Cam (1:1 v/v).

Other variables related to the extraction step were studied, including extraction time (17–110 min) and sample pH (5–13) which were evaluated using a central composite design. The response surface obtained through this optimization is shown in Figure 3 considering the geometric means of the chromatographic peak area for the analytes.

The response surface exhibited *R* ^2^ = 0.9023 which consists of adequate fitting for the statistical model. According to Figure 3, longer extraction times and pH ranging from 12 to 13 provided the best chromatographic responses for the analytes. In this regard, pH at 12 was selected for further experiments. Related to the extraction time, considering the extraction efficiency and sample throughput, 100 min was selected as it provided satisfactory results in terms of chromatographic peak area. Importantly, the use of a multiwell platform capable of processing 96 samples simultaneously is an important feature of this experimental workflow.

### 3.3 Analytical Parameters of Merit and Analysis of Real Samples

After all optimization steps, the analytical parameters of merit of the HF‐MMLLE approach were determined. In this case, calibration curves were performed with five concentrations of each analyte using the chromatographic peak areas as analytical response. The analytical parameters are shown in Table 1.

**TABLE 1:** Analytical figures of merit for the validation of hollow fiber‐microporous membrane liquid–liquid extraction (HF‐MMLLE) methodology for the three hormones studied.

Analyte	LOD (ng mL^−1^)	LOQ (ng mL^−1^)	*R* ^2^	Linear range (ng mL^−1^)	Linear Equation	Concentration (ng mL^−1^)	Precision		Relative recovery (%)	
							Intraday (*n* = 3)	Interday (*n* = 9)	Sample A	Sample B
17β‐Estradiol	15.0	50.0	0.9913	50.0–500.0	*y* = 536.9*x* − 6220.8	50.0	4.6	21.6	72.4 ± 3.2	119.7 ± 7.0
						300.0	1.5	16.5	68.2 ± 3.3	124.4 ± 3.9
						500.0	6.1	13.8	75.5 ± 9.2	130.5 ± 27.7
Estrone	7.5	25.0	0.9926	25.0–500.0	*y* = 423.2*x* + 447.4	50.0	2.2	9.7	93.5 ± 9.8	118.7 ± 10.4
						300.0	1.1	10.9	99.8 ± 10.3	126.5 ± 3.9
						500.0	2.7	8.5	109.6 ± 7.2	127.2 ± 4.5
17α‐Ethinylestradiol	15.0	50.0	0.9931	50.0–500.0	*y* = 390.2*x* − 5345.9	50.0	8.7	19.1	76.9 ± 3.1	110.9 ± 10.7
						300.0	0.2	12.2	78.4 ± 10.5	126.9 ± 2.8
						500.0	5.4	8.9	96.2 ± 11.5	131.8 ± 30.2

*Note*: Experimental conditions: desorption time of 30 min; ACN as desorption solvent; extraction time of 100 min; pH adjusted to 12; NADES—Thyl:Cam (1:1 v/v).

Abbreviations: LOD, limit of detection; LOQ, limit of quantification; NADES, natural deep eutectic solvent.

According to Table 1, linear ranges varied from 25 to 500 ng mL^−1^ with coefficients of determination ≥0.9926, indicating a satisfactory linearity of the calibration curves. LOQs were established as the first point of the calibration curves, consisting of 25 ng mL^−1^ for E2, and 50 ng mL^−1^ for E1 and EE2. LODs were calculated on the basis of LOQ divided by 3.3, corresponding to 7.5 ng mL^−1^ for E2, and 15 ng mL^−1^ for E1 and EE2. Precision was evaluated at three concentrations (50, 300, and 500 ng mL^−1^) consisting of intraday (*n* = 3) and interday (*n* = 9) with values ranging from 0.2% to 8.7% and 8.5% to 33.6%, respectively. Relative recovery was examined in two urine samples obtained from volunteers spiked at three concentrations (50, 300, and 500 ng mL^−1^) and subjected to the experimental workflow developed in this study. Sample A exhibited results from 68.2% to 109.2%, and sample B presented recoveries from 110.9% to 131.8%. In this regard, satisfactory results were obtained in different samples of urine being in accordance with validation guidelines [36].

In clinical terms, several studies indicate low concentrations of both compounds in healthy men and women. For example, in children (male and female), the concentrations of natural hormones (E1 and E2) should be in the range of pg mL^−1^ in the blood, and consequently, when excreted in the urine, they should be in these same concentrations or lower. Adult women who are pre‐ and postmenopausal have low levels of these compounds, on the scale of pg mL^−1^ to ng mL^−1^ naturally, including adult men (pg mL^−1^), and for women in the gestation stage, the levels become very high due to the entire process of modification of the organism for the generation of a baby, being concentrations above 2 ng mL^−1^. For EE2, the presence should not be detected in children, during pregnancy in women, and in the postmenopausal period, unless they are taking hormonal therapy. In any situation in which contraceptive medicine containing EE2 is not being used, sources of contamination should be detected due to the high levels that it will present in the urine (0.020 ng mL^−1^ to levels in µg mL^−1^) [4, 37, 38, 39, 40, 41, 42]. In this context, considering the levels obtained in this work, on the ng mL^−1^ scale, it is used to detect these hormones at high levels in the body that are directly related to disorders, whether endocrine or pregnancy, and, as one of the main focuses, the development of breast and ovarian cancers, as well as the presence of endometriosis in women.

The HF‐MMLLE approach was applied in five samples obtained from healthy women that declared to make use of contraceptives. It is worth noting that these hormones can also be present in the female body in small concentrations. Chromatograms obtained through extractions using this methodology are presented in Figure S3. Chromatograms of Figure S3 (A01–A05) correspond to extractions from real urine samples obtained from female volunteers, aged from 18 to 32. Moreover, Figure S3 (A06) is a chromatogram obtained from a direct injection of the standards E1, E2, and EE2. The chromatogram A07 of Figure S3 represents the combination of all samples analyzed. It is possible to observe a peak corresponding to E2 found in all samples. Regarding samples A01, A02, and A03, it was possible to quantify E2 at concentrations of 182.7, 180.36, and 75.6 ng mL^−1^, respectively. Related to the samples A04 and A05, the chromatographic peaks were detected but the concentrations were below the LOQ (25 ng mL^−1^). The other compounds were not detected in the samples analyzed. It is worth mentioning that E2 can be naturally found at concentration levels of ng mL^−1^ in women, which indicates acceptable concentrations according to the literature, indicating no changes associated with health problems [37, 38, 39, 40, 41, 42].

### 3.4 Comparison of the Methodology With Previously Reported Studies

Finally, some of the analytical features of the methodology developed in this study were compared with previous studies reported in the literature. Table 2 contains aspects related to each approach for the steroidal hormones examined in urine samples.

**TABLE 2:** Comparison of the proposed method with studies reported in the literature for the determination of the hormones in urine samples.

Analytes	LOQ (ng mL^−1^)	Sample preparation technique	Extraction phase	Sample volume	Average sample preparation time/per sample (min)^a^	Instrumentation	Ref.
E1 E2 EE2	50.0 25.0 50.0	HF‐MMLLE	NADES (Cam:Thy 1:1 molar ratio)	1.5 mL	∼1.45	HPLC‐DAD	This work
E1 E2	50.0	DLLME	Acetonitrile and chloroform	3 mL	∼30	UHPLC‐HRMS	[46]
E1 E2 EE2	40.0 4.0 20.0	TF‐SPME	Bract	1.5 mL	∼2.4	HPLC‐FLD	[5]
E1 EE2	20.0	DLLME	[P_6,6,6,14_^+^]_2_[MnCl_4_^2−^]	150 µL	∼11	HPLC‐DAD	[47]
E1 E2 EE2	15.0 0.1 1.0	HF‐MMLLE	1‐Octanol	1.5 mL	∼0.6	HPLC‐FLD	[9]
E1 E2 EE2	1.0 5.0 10.0	TF‐SPME	PAni‐silica doped with oxalic acid	1.5 mL	∼2.0	HPLC‐FLD	[48]
E1	0.5	SPE	Strata C‐18‐E Cartridge	1 mL	∼60	HPLC‐Q‐TOF	[49]

*Note*: Experimental conditions used in this work: desorption time of 30 min; ACN as desorption solvent; extraction time of 100 min; pH adjusted to 12; NADES—Thyl:Cam (1:1 v/v).

Abbreviations: HF‐MMLLE, hollow fiber‐microporous membrane liquid–liquid extraction; HPLC‐DAD, high‐performance liquid chromatography coupled with a diode array detector; LOQ, limit of quantification; NADES, natural deep eutectic solvent; SPE, solid‐phase extraction.

^a^ Total sample preparation time divided by the number of samples being processed.

According to Table 2, this methodology featured similar LOQs to those obtained with previously reported methodologies. Importantly, HPLC‐DAD used in this study provided satisfactory analytical performance associated with lower costs compared to more sophisticated instruments such as UHPLC‐HRMS and HPLC‐Q‐TOF which also have been used for determining these analytes. The HF‐MMLLE technique is relatively simple to be coupled with a 96‐well plate system featuring the capacity of processing multiple samples. This fact consists of an important advantage of this methodology as the sample preparation time is less than 2 min per sample considering the system operating at full capacity (96 samples simultaneously), offering a high‐throughput approach that can be applied for routine analysis. Another important aspect in terms of sustainability is the use of natural and biodegradable solvents such as NADES [43, 44, 45]. In this regard, a safe and environment‐friendly sample preparation approach was demonstrated combining satisfactory analytical performance with low waste generation.

### 3.5 Sustainability Assessment

**FIGURE 4:**
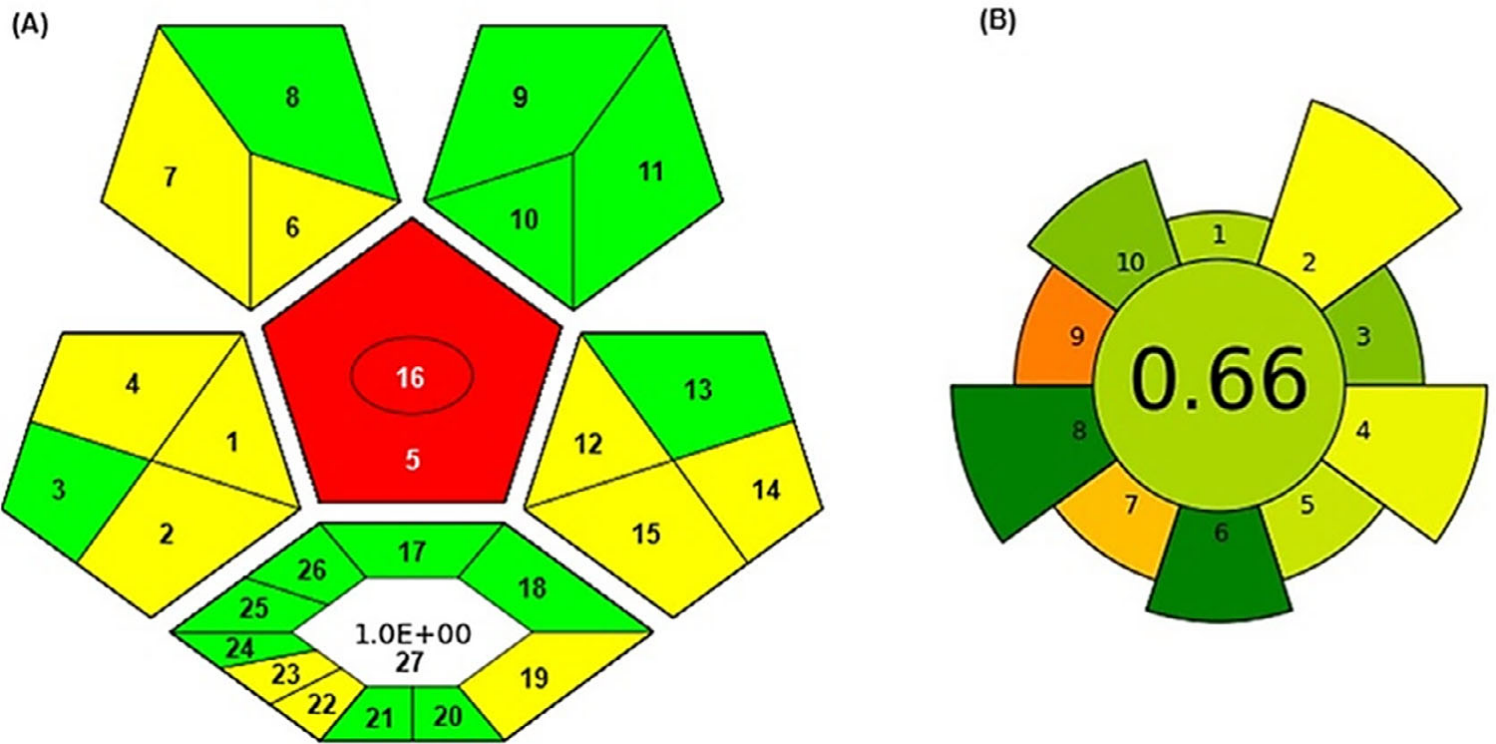
Evaluation of the sustainable aspects of the methodology using (A) ComplexGAPI and (B) AGREEprep.

This method was subjected to sustainability assessment using some recently proposed metrics, including the ComplexGAPI and AGREEprep [32, 33]. For GAPI, the entire analytical methodology is considered, also including parameters involved in pre‐analysis, sample preparation, and instrumental analysis. AGREEprep, which is a derived tool from AGREE [50], focuses on the parameters directly involved in the sample preparation step, being further subdivided into specific elements. The pictograms are presented in Figure 4.

Evaluating the pictogram obtained by the ComplexGAPI shown in Figure 4A, the proposed method demonstrates predominantly green and yellow zones, indicating a procedure with low to moderate environmental impact. This distribution reflects a sustainable analytical workflow, where most steps agree with GAC principles. The presence of green areas highlights minimal use of hazardous reagents, reduced waste generation, and energy efficiency, whereas the yellow parts suggest further improvements in terms of environmental performance [32].

Figure 4B shows the pictogram generated with the AGREEprep tool. The score obtained for this study was 0.66, with green and yellow regions, indicating that the method also featured some sustainable aspects [33]. This result highlights that the proposed method incorporates several sustainable features in the sample preparation stage. In particular, the favorable score is attributed to key aspects such as low consumption of sample and solvents, enhanced sample throughput, minimal waste generation, and the use of safer materials and procedures. These aspects emphasize the concerns with GAC principles, making this method a sustainable alternative for the determination of steroidal hormones in urine samples.

## 4 Conclusion

A sustainable HF‐MMLLE approach based on NADES was successfully combined with a 96‐well plate system for the determination of steroid hormones in human urine by HPLC‐DAD. The methodology was comprehensively optimized and validated for the determination of 17β‐estradiol (E1), estrone (E2), and 17α‐ethinylestradiol (EE2) which are key endocrine‐disrupting compounds and recognized as potential biological markers for pathologies such as breast and uterine cancer and for the diagnosis of endometriosis. Robust analytical performance and high sample throughput were achieved with this experimental workflow, including precision, accuracy, sensitivity, and linearity across clinically relevant concentration ranges, assuring its applicability in clinical and research purposes. The use of NADES as extraction media highlights the environmental aspects of the methodology. These solvents are composed of natural, biodegradable, and low‐toxicity components, reducing the environmental impact associated with conventional organic solvents. This fact aligns the developed method with several of the United Nations Sustainable Development Goals (SDGs), especially those targeting responsible consumption and production, good health and well‐being. Moreover, the application of the 96‐well plate format enabled increased sample throughput, making the method suitable for large‐scale or routine bioanalytical studies. However, despite the high degree of miniaturization and partial automation, certain procedural steps still necessitate manual intervention and monitoring by trained analysts. Therefore, although the system can be considered semiautomated, further efforts could be directed toward fully automating the workflow to enhance efficiency and reduce operator dependency. The software used for the green evaluation declared that the method presents sustainability and aspects aligned with the GAC, particularly in terms of time, solvent safety, waste minimization, and energy efficiency. The entire concept and development of the methodology were aligned with providing a bioanalytical method that was satisfactory in high concentration ranges of these hormones, determining a possible anomaly in the organism.

## Author Contributions

**Lucas Morés**: conceptualization, methodology, validation, investigation, writing – original draft. **Camila Will**: writing – review and editing, validation. **Eduardo Carasek**: supervision, resources, writing – review and editing. **Josias Merib**: writing – review and editing, resources, supervision, project administration.

## Conflicts of Interest

The authors declare no conflicts of interest.

## Supporting information




**Supporting File 1**: jssc70229‐sup‐0001‐SuppMat.docx.
