# Supplementary material for: A Membrane‐Based Strategy for the High‐Throughput Determination of Steroidal Hormones in Human Urine Using Natural Deep Eutectic Solvents Combined With Liquid Chromatography Coupled With Diode Array Detector
Source: J Sep Sci. 2025 Jul 17;48(7):e70229. doi: 10.1002/jssc.70229 (PMC12271989; doi:10.1002/jssc.70229)

**Supplementary Material**

**A Membrane-Based Strategy for the High-Throughput Determination of Steroidal Hormones in Human Urine Using Natural Deep Eutectic Solvents Combined with Liquid Chromatography Coupled with Diode Array Detector**

Lucas Morés^a,b,c^, Camila Will^c^, Eduardo Carasek^c^, Josias Merib^a,b*^

^a^ Departamento de Farmacociências, Universidade Federal de Ciências da Saúde de Porto Alegre, Porto Alegre, RS, 90050-170, Brazil.

^b^ Programa de Pós-Graduação em Biociências, Universidade Federal de Ciências da Saúde de Porto Alegre, Porto Alegre, RS, 90050-170, Brazil.

^c^ Departamento de Química, Universidade Federal de Santa Catarina, Florianópolis, SC, 88040-900, Brazil.

*Corresponding Author (J. Merib)

E-mail address: [josias@ufcspa.edu.br](mailto:josias@ufcspa.edu.br)

**Table 1-S:** Compounds used in NADES formations to be studied in this work.

|  | **Compound 1** | **T_f_ (°C)** | **Compound 2** | **T_f_ (°C)** | **Molar Ratio** | **Abbreviation** |
| --- | --- | --- | --- | --- | --- | --- |
| 1 | Dodecanoic Acid | 44.85 | Octanoic Acid | 15.85 | 1:1 | C12:C8 |
| 2 | Dodecanoic Acid | 44.85 | Dodecanoic Acid | 31.85 | 1:1 | C12:C10 |
| 3 | Dodecanoic Acid | 44.85 | Hexanoic Acid | -2.15 | 1:1 | C12:C6 |
| 4 | Dodecanoic Acid | 44.85 | Butyric Acid | -7.9 | 1:1 | C12:C4 |
| 5 | Dodecanoic Acid | 44.85 | Nonanoic Acid | 11.85 | 1:2 | C12:C9 |
| 6 | Decanoic Acid | 31.85 | Hexanoic Acid | -2.15 | 1:3 | C10:C6 |
| 7 | Decanoic Acid | 31.85 | Camphor | 174-179 | 1:1 | C10:Ca |
| 8 | Decanoic Acid | 31.85 | Thymol | 49.6 | 1:1 | C10:T |
| 9 | Thymol | 49.6 | Camphor | 174-179 | 1:1 | T:Ca |
| 10 | Thymol | 49.6 | Levulinic Acid | 33 | 1:1 | T:L |

**Figure 1-S:** Bar graph of the NADES applied for the extraction of hormones in ultrapure water: (1) C12:C8; (2) Thymol:C10; (3) Ca:C10; (4) L:T; (5) C12:C10; (6) C12:C6; (7) C12:C4; (8) T:Ca; (9) C12:C9; (10) C10:C6; (11) No NADES. Experimental conditions: urine adjusted to pH 10; extraction time of 60 min; desorption time of 20 min; acetonitrile as desorption solvent; analytes spiked at 500 µg L^-1^.

**Figure 2-S:** Bar graph obtained for the desorption time evaluation.

Experimental conditions: Urine adjusted to pH 11; extraction time of 60 min; DES - Thymol:Camphor (1:1) as extraction solvent; acetonitrile as desorption solvent; and analytes spiked at 500 µg L^-1^.

**Figure 3-S:** Chromatograms from extractions performed in urine samples obtained from volunteers (A01-A05); a chromatogram from an extraction performed in blank urine sample spiked at 500 μg L^-1^ (A06). The chromatogram (A07) consists of all chromatograms (A01 to A05) overlapped. In this case, the green line is sample A01; the black line is sample A02; the dark blue line is sample A03; the pink line is sample A04; and the blue line sample A05. (Experimental Conditions: desorption time of 30 min; acetonitrile as desorption solvent; extraction time of 100 min; pH adjusted to 12; analytes spiked at 500 ng mL^-1^; NADES - Thyl:Cam (1:1 v/v).


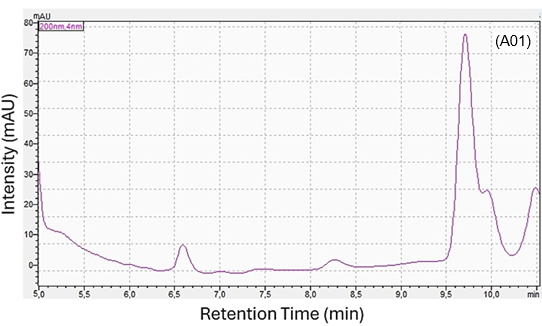

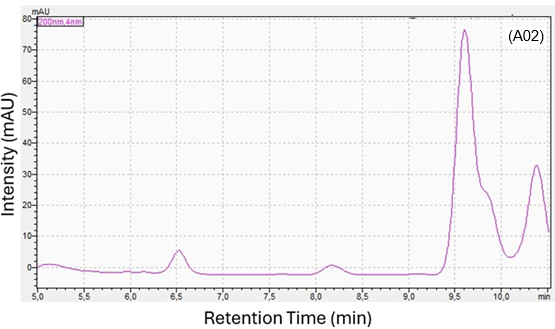


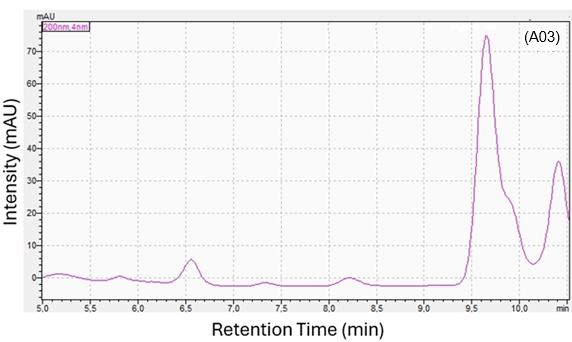

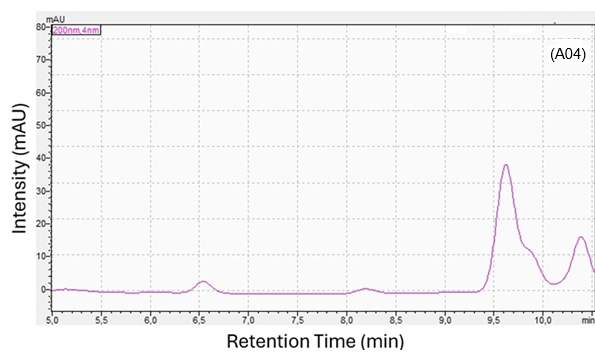

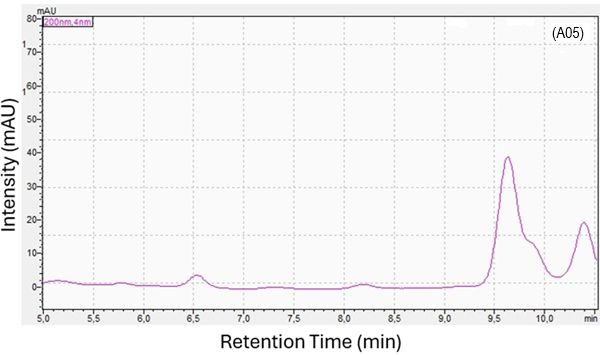

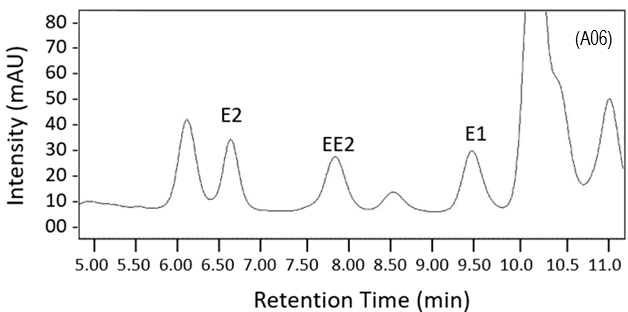


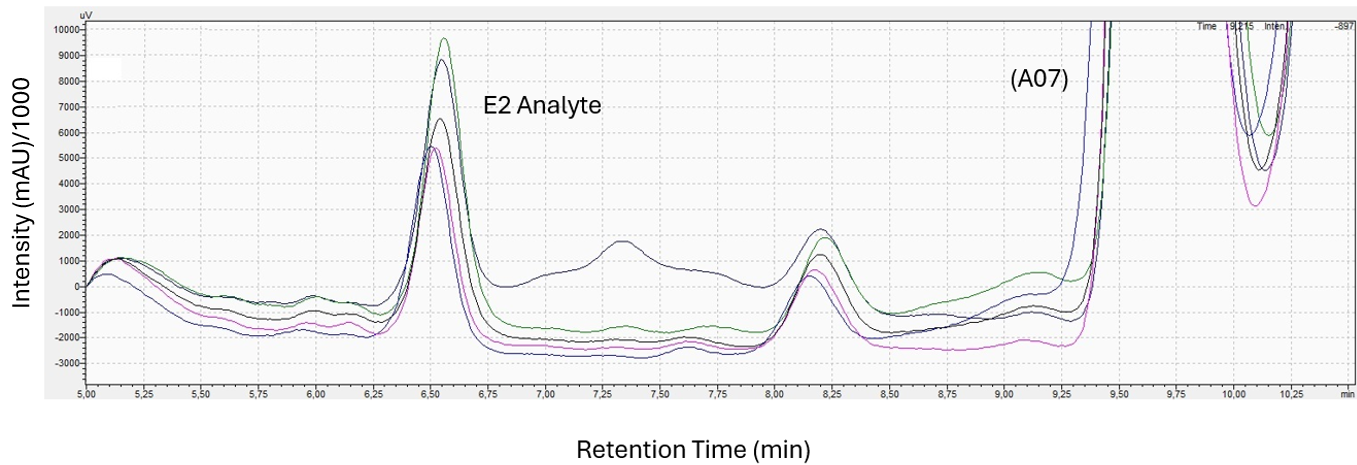

Supplement: Supplementary file 1 — Supporting File 1: jssc70229‐sup‐0001‐SuppMat.docx. [file JSSC-48-e70229-s001.docx]
